# Effect of supplementing honey via drinking water on growth performance, carcass traits, and blood biochemical parameters in broiler chickens

**DOI:** 10.5194/aab-69-11-2026

**Published:** 2026-01-07

**Authors:** Soha A. Farag, Ahmed K. Aldhalmi, Vincenzo Tufarelli, Waleed M. Dosoky, Ayman A. Swelum, Abdulmohsen H. Alqhtani, Hanan M. Alharbi, Khairiah M. Alwutayd, Antonella Perillo, Caterina Losacco, Mohamed E. Abd El-Hack, El-Kazafy A. Taha

**Affiliations:** 1 Department of Animal Production, Faculty of Agriculture, Tanta University, Tanta 31527, Egypt; 2 College of Pharmacy, Al-Mustaqbal University, 51001 Babylon, Iraq; 3 Department of Precision and Regenerative Medicine and Jonian Area, Section of Veterinary Science and Animal Production, University of Bari Aldo Moro, 70010 Valenzano, Bari, Italy; 4 Department of Animal and Fish Production, Faculty of Agriculture Saba Basha, Alexandria University, Alexandria 21531, Egypt; 5 Department of Animal Production, College of Food and Agriculture Sciences, King Saud University, Riyadh 11451, Saudi Arabia; 6 Department of Biology, College of Science, Princess Nourah bint Abdulrahman University, P.O. Box 84428, Riyadh 11671, Saudi Arabia; 7 Department of Industrial Pharmacy, College of Pharmaceutical Sciences and Drug Manufacturing, Misr University for Science and Technology (MUST), Giza, Egypt; 8 Poultry Department, Faculty of Agriculture, Zagazig University, Zagazig 44511, Egypt; 9 Department of Economic Entomology, Faculty of Agriculture, Kafrelsheikh University, Kafrelsheikh 33516, Egypt

## Abstract

This study investigated the effects of supplementing broiler drinking water with Egyptian clover honey on growth performance, carcass characteristics, and blood biochemical parameters. A total of 525 one-day-old male Hubbard broilers were assigned to seven treatment groups to evaluate two concentrations of honey (13 and 26 $\mathrm{mL}\;L^{-1}$ ) administered at three different frequencies (daily, every other day, and every 3 d), along with a control group receiving no honey. Supplementing drinking water with honey significantly enhanced overall broiler performance. Birds receiving honey, particularly at the higher concentration and at intermittent intervals, showed improved body weight gain and feed efficiency compared to the control group. The highest relative weights of key lymphoid organs – such as the spleen, bursa of Fabricius, and thymus – were observed in supplemented groups, indicating a positive impact on immune system development. Hematological analysis revealed increased red and white blood cell counts, hemoglobin, and packed cell volume in honey-fed birds, reflecting improved oxygen transport and immune competence. Serum biochemical profiles demonstrated a favorable shift, with significant reductions in markers of metabolic stress and liver function (uric acid, creatinine, cholesterol, triglycerides, and alanine aminotransferase (ALT)) and notable increases in total protein, albumin, and globulin levels. Additionally, honey supplementation significantly enhanced humoral immune response, as evidenced by higher hemagglutination inhibition titers against Newcastle disease virus. The results indicate that honey supplementation via drinking water, especially at 26 $\mathrm{mL}\;L^{-1}$ , administered intermittently, can effectively improve growth performance, physiological health, and immune function in broiler chickens. These findings support the potential of honey as a natural antibiotic-free additive to promote sustainable poultry production.

## 1 Introduction

Using natural products is encouraged in poultry production to improve growth performance. Some products of beehives have been supplemented in poultry diets, e.g., bee pollen (El-Deeb et al., 2024), honey (Japhet and Oyingebarakumo, 2021), bee venom (Abd El-Aziz et al., 2023; Kim et al., 2019), propolis (Abbas et al., 2020; Al-Kahtani et al., 2022; Prakatur et al., 2019), and royal jelly (Abd El-Aziz et al., 2023). In this context, Japanese quail has been shown to exhibit reduced oxidative stress when supplemented with a propolis diet. Also, adding bee pollen to broiler diets has promoted some immunological traits, including a reduction in heterophil to lymphocyte ratio, an increase in antibody production, an increase in leukocytes, and a reinforcement of immune-organ formation in broilers (Farag and El-Rayes, 2016). Furthermore, several investigations have proved that broiler chicks and Japanese quails improved in growth performance, hematological analysis, and immune functions through dietary propolis and/or bee pollen supplementation (Al-Kahtani et al., 2022; Dosoky et al., 2016).

Honey has gained growing interest in poultry nutrition as producers seek natural residue-free alternatives to antibiotics and synthetic growth promoters. Unlike broader bee products such as propolis, pollen, or royal jelly, which each have distinct bioactive profiles, honey offers a unique combination of readily available sugars, organic acids, enzymes, antioxidants, minerals, and antimicrobial compounds that can directly influence gut health, metabolism, and immune function in poultry (Taha et al., 2018). Its diverse bioactive matrix makes honey more than an energy source; it acts as a multifunctional natural additive that supports physiological and productive performance (Japhet and Oyingebarakumo, 2021). One of the most relevant contributions of honey is its prebiotic-like effect. The presence of oligosaccharides, glucose oxidase, and low pH environment can help to stimulate beneficial gut microbiota while suppressing pathogenic bacteria (Abd El-Aziz et al., 2023). This microbial modulation results in improved nutrient absorption, reduced intestinal stress, and an enhanced villi structure, all of which contribute to better feed conversion and growth rates. Compared with other bee-derived products, honey is generally more palatable, easier to incorporate into feed or drinking water, and can be rapidly utilized by the digestive system, giving honey a practical advantage in commercial production settings (Taha et al., 2018).

The physicochemical properties of honey have been previously studied (Taha et al., 2018, 2021). They depend mainly on the botanical origin (Solayman et al., 2016), geographical origin (Taha et al., 2017; Eissa and Taha, 2023), species or subspecies of honey bee (Taha et al., 2021), storage-time length (Al-Ghamdi et al., 2019), and comb age (Taha et al., 2010). The standard chemical characteristics of honey have been described in Codex Alimentarius (Codex Alimentarius Commission, 2001) as ≤20% moisture, >60% glucose and fructose, ≤5% sucrose, pH 3.40–6.10, and ≤50 $\mathrm{mmol}\;{\mathrm{kg}}^{-1}$ acidity. In addition, honey contains other carbohydrates, mineral elements, enzymes, aromatic substances, amino and organic acids, and pollen grains (Taha et al., 2021; Boussaid et al., 2018).

Honey is widely recognized for its remarkable antioxidant properties, offering protection against major health problems such as heart disease, cataracts, immune deficiencies, cancer, and inflammation (Molan and Betts, 2004). The growing concern about the negative impacts of synthetic drugs on human well-being has further encouraged the search for natural alternatives (Can et al., 2015). Because foods rich in antioxidants can help to prevent and manage chronic diseases by acting as internal cellular defenders against free radicals (Bozdogan Konuskan and Mungan, 2016), honey has gained increasing scientific attention. It is considered a nutrient-dense natural product with strong antioxidant capacity (Gül and Pehlivan, 2018; Stefanis et al., 2023) and is known for its broad biological activities, including antimicrobial, anticancer, antimutagenic, anti-inflammatory, immunological, antiparasitic, and antiviral effects (Gül and Pehlivan, 2018; Bogdanov et al., 2008; Asma et al., 2022; Feknous and Boumendjel, 2022). However, despite its diverse health benefits, its application in poultry production remains surprisingly underexplored.

Recent insights highlight the fact that honey's flavonoids and phenolic acids can significantly reduce oxidative stress – an issue commonly faced by fast-growing broilers and birds exposed to heat stress or high stocking density (Taha et al., 2018). Moderate dietary honey supplementation has been associated with enhanced antibody titers, improved lymphoid organ activity, and greater resistance to infections, making it a promising tool in antibiotic-reduction strategies where sustaining immune competence is vital (Abd El-Aziz et al., 2023; Eissa and Taha, 2023). Beyond immunity, honey may support metabolic regulation; its simple sugars provide rapid energy that aids early chick development and recovery after stress. Meanwhile, bioactive molecules such as hydrogen peroxide and methylglyoxal offer antimicrobial protection, helping to lower the risk of enteric disturbances that often hinder growth performance (Eissa and Taha, 2023).

Although honey is widely recognized for its valuable bioactive properties, its use specifically in drinking water for broilers has been far less studied compared with other bee-derived products. This gap is noteworthy because honey is a clean, natural, and practical additive that aligns well with today's growing demand for naturally produced poultry. While honey is known to support growth, gut health, immunity, and antioxidant defenses, there is still limited evidence on how delivering it through drinking water influences broiler performance and physiological responses. This is important to explore, as water-based supplementation may provide a more uniform and efficient way for birds to consume bioactive compounds compared to feed inclusion.

The novelty of the present study lies in its focused evaluation of honey as a drinking water supplement, simultaneously assessing both concentration and dosing frequency – an aspect not previously explored in research on broiler nutrition. In addition, the study provides a comprehensive investigation of honey's effects on growth performance, blood parameters, biochemical markers, and immune responses, aiming to offer new insights that extend beyond existing feed-based honey studies. Accordingly, we examined whether supplementing broiler drinking water with honey can lead to measurable improvements in both productivity and overall physiological health.

## 2 Materials and methods

### Chemical characteristics of honey 

Egyptian clover (*Trifolium alexandrinum* L.) honey was obtained from the apiary of the Faculty of Agriculture, Kafrelsheikh University, Kafrelsheikh, Egypt. Moisture, ash, and crude protein contents were determined using the Association of Official Analytical Chemists (AOAC, 2000) methods. The method of AOAC (AOAC, 2000) was used to estimate fructose, glucose, sucrose, and maltose by utilizing the HPLC chromatographic. A spectrophotometric Folin–Ciocalteu method was used to determine the total phenols (Singleton et al., 1999). White et al. (1962) determined free acidity and total acidity content. A glass rod Hanna pH meter, model H 19321 was used to examine the pH value (AOAC, 2000).

### Macro- and trace element determination

A 1 g honey sample was wet-digested with nitric acid following the AOAC (AOAC, 2000) protocol to prepare it for macro- and trace element analysis. We used an atomic absorption spectrophotometer (GBC Avanta E, serial no. A5616) to determine the concentrations of calcium (Ca), sodium (Na), potassium (K), iron (Fe), magnesium (Mg), copper (Cu), zinc (Zn), and manganese (Mn). To detect phosphorus (P), a UV-VIS spectrophotometer (UV-2550 Shimadzu, serial no. A108447) was used.

### Birds and experimental design

The Ethics Committee of Tanta University, Egypt, approved all experimental procedures, with reference number for the ethical approval AY2019-2020/Session6/2020.01.13.

A total of 525 one-day-old male Hubbard broilers were individually weighed and wing-banded, then randomly assigned to seven equal treatment groups. Each group was further divided into three replicates, with 25 broilers per replicate. The first group received drinking water (DW) without additives, and the second, third, and fourth groups received honey in DW (13 mL per L water daily), day after day (every other day), and day after 2 d (once every 3 d), respectively. The fifth, sixth, and seventh groups received honey in DW at 26 mL per L water daily, day after day (every other day), and day after 2 d (once every 3 d), respectively. These dosing schedules were designed to determine whether varying the frequency of honey administration from continuous to intermittent could sustain its physiological and microbial benefits while offering practical cost-effective strategies for poultry producers. Feed and water were provided ad libitum until the end of the experiment. The broilers were fed on starter and grower diets from 1–21 and 22–35 d of age, respectively. The experimental diets were formulated to be isocaloric and isonitrogenous, according to the National Research Council (NRC, 1994). The chemical analysis of experimental diets is presented in Table 1. The broilers were vaccinated against Newcastle disease (ND) using Hitchiner B1 on the seventh day and Lasota vaccine on the 18th and 28th days in DW. Also, they were vaccinated against Gumboro disease using Gumboro D78 in DW on days 14 and 21. The chicks were housed in separate floor pens bedded with wood shavings and kept exposed to 23 h of light and 1 h of darkness throughout the experimental period. To ensure optimal welfare and uniform growth conditions, the environmental parameters were carefully controlled. Temperature was maintained at 32–34 °C during the first week and gradually lowered to 24–26 °C , while relative humidity remained between 55 % and 65 %. Pens were managed at a stocking density of 10 birds per $m^{2}$ and equipped with continuous mechanical ventilation to provide consistent air movement so as to maintain air quality.

**Table 1:** Composition and calculated analysis of experimental basal diet during starter and grower stages.

Ingredients		Starter	Grower
		(1–21 d)	(22–35 d)
Yellow corn		50.47	57.99
Soybean meal (44 % CP)		32.55	30.88
Corn gluten meal		7.11	2.5
Soybean oil		6.0	5.5
Limestone		1.47	1.33
Dicalcium phosphate		1.67	1.16
NaCl		0.3	0.29
Premix*		0.3	0.3
Dl-methionine		0.11	0.05
L-lysine		0.02	–
Calculated analysis			
	Crude protein (%)	23.02	20.07
	Metabolizable energy ( $\mathrm{kcal}\;{\mathrm{kg}}^{-1}$ )	3200	3200
	Ether extract (%)	2.36	2.51
	Crude fiber (%)	3.48	3.47
	Calcium (%)	1.03	0.86
	Available phosphorus (%)	0.45	0.35
	Methionine (%)	0.51	0.38
	Lysine (%)	1.10	1.00

* Each 3 kg of premix contained vitamin A, 123 000 IU; vitamin D3, 2500 IU; vitamin E, 10 mg ; vitamin K3, 2000 mg ; vitamin B1, 1000 mg ; vitamin B2, 5000 mg ; vitamin B6, 1500 mg ; vitamin B12, 10 mg ; niacin, 30 mg ; biotin, 50 mg ; folic acid, 1000 mg ; pantothenic acid, 10 mg ; manganese, 60 mg ; zinc, 50 mg ; iron, 30 mg ; copper, 4000 mg ; iodine, 300 mg ; selenium, 100 mg ; and cobalt, 100 mg .

### Growth performance and carcass traits

The body weight (BW), body weight gain (BWG), feed consumption (FC), and feed conversion ratio (FCR) were determined weekly. Water consumption was recorded daily. The number of dead broilers was recorded on the day when the bird died. The viability rate was estimated using the following equation: viability rate = (no. alive birds/no. initial birds) ×100 . On the 35th day of age, six chickens of each treatment were randomly chosen, weighed, and slaughtered. The carcass, gizzard, heart, liver, spleen, bursa, and thymus were weighed, and the relative percentage of BW was calculated.

### Blood parameters

At 35 d, two broilers of each replicate (six broilers per treatment) were used for blood samples. Two samples were collected from the wing vein immediately before slaughtering. The first sample was collected in heparinized test tubes to determine the hematological parameters. Packed cell volume (PCV) was measured using the microhematocrit method, according to Benson et al. (1989). The hemoglobin (Hb) concentration was estimated. Red blood cell (RBC) and white blood cell (WBC) counts were estimated by using the hemocytometer method (Lamb, 1981), while the differential leucocyte (heterophil, lymphocyte, eosinophil, monocyte, and basophil) counts (Schalm, 1986) heterophil to lymphocyte (H / L) ratio was estimated. The second blood sample was used to separate the blood serum by centrifuging at 4000 rpm for 15 min . The obtained serum was frozen at -20 °C until analyzed to determine the uric acid (Trinder, 1969), creatinine (Fabiny and Ertingshausen, 1971), total protein (Henry, 1964), albumin (Doumas et al., 1971), cholesterol (Allina et al., 1974), triglyceride (Sidney and Barnard, 1973), serum alanine aminotransferase (ALT), and aspartate aminotransferase (AST) (Reitman and Frankel, 1957) by a spectrophotometer (Spectronic 1201, Milton Roy, Ivyland, PA, USA) using commercial kits (AUTOPAK, Bayer Diagnostics, India) according to the manufacturer's instructions. The serum globulin was calculated by subtracting albumin from total protein. Hemagglutination inhibition (HI) test against Newcastle disease (ND) was determined in broilers' serum at the age of 35 d (Kaleta and Siegmann, 1971).

### Statistical analysis

Data were analyzed using the general linear model (GLM) procedure in SAS software version 9.1 (SAS Institute Inc., Cary, NC, USA) (SAS Institute, 2003). The experiment followed a 2×3 factorial design, examining honey concentration (13 or 26 $\mathrm{mL}\;L^{-1}$ ) and administration frequency (daily, every other day, or every third day), along with an additional control group that received no honey. The pen ( n=3 per treatment) was considered to be the experimental unit for all analyses. The statistical model used was $Y_{ijk}=\mu +C_{i}+F_{j}+(C\times F{)}_{ij}+\varepsilon_{ijk}$ , where $Y_{ijk}$ is the observed value for the dependent variable, μ is the overall mean, $C_{i}$ is the fixed effect of honey concentration ( $i=0,13,26\;\mathrm{mL}\;L^{-1}$ ), $F_{j}$ is the fixed effect of administration frequency ( j= daily, day after day, day after 2 d), $(C\times F{)}_{ij}$ is the interaction effect between concentration and frequency, and $\varepsilon_{ijk}$ is the random error term associated with the k th replicate of the i th concentration and j th frequency.

To evaluate the effects of honey concentration, administration frequency, and their interaction, F tests were used. The Shapiro–Wilk test was applied to check for normality, and Levene's test verified homogeneity of variances. After confirming these assumptions, the data were analyzed. When a significant F test was observed ( P<0.05 ), Duncan's multiple range test was used to compare the least-squares means of the seven treatment groups. Differences were considered statistically significant at P<0.05 .

## 3 Results

### Chemical characteristics of the honey

The data in Table 2 show that the concentration of moisture, sucrose, fructose, glucose, maltose, crude protein, and ash in the Egyptian clover honey were 18.03 %, 40.44 %, 32.11 %, 2.74 %, 0.76 %, and 0.34 %, respectively. Egyptian clover honey had 1.88 mg per 100 g total phenols, a pH of 3.35, and 31.54 $\mathrm{mg}\;{\mathrm{kg}}^{-1}$ free acidity. The amounts ( $\mathrm{mg}\;{\mathrm{kg}}^{-1}$ ) of K, Ca, P, Na, Mn, Zn, Fe, Mg, and Cu were 760.45, 608.12, 592.71, 584.76, 0.53, 2.29, 36.84, 441.33, and 0.12 $\mathrm{mg}\;{\mathrm{kg}}^{-1}$ , respectively (Table 3).

**Table 2:** The chemical characteristics of the honey.

Component	
Moisture %	18.03
Fructose %	40.44
Glucose %	32.11
Maltose %	3.66
Sucrose %	2.74
Crude protein %	0.76
Ash %	0.34
Total phenols, mg per 100 g	1.92
pH	3.35
Free acidity, $\mathrm{mq}\;{\mathrm{kg}}^{-1}$	31.54
Total acidity, $\mathrm{mq}\;{\mathrm{kg}}^{-1}$	41.88

**Table 3:** Macro- and trace elements of the honey.

Mineral	$\mathrm{mg}\;{\mathrm{kg}}^{-1}$
K	760.45
Ca	608.12
P	592.71
Na	584.76
Mg	441.33
Fe	36.84
Zn	2.29
Mn	0.53
Cu	0.12

### Growth performance 

The live BW and BWG of broiler chicks were significantly ( P<0.01 ) influenced by adding honey to DW (Table 4). At 7 and 14 d of age, significant ( P<0.01 ) differences were detected between the BW of broilers receiving the honey day after 2 d either at a level of 13 or 26 mL per L water and other treatments. Meanwhile, insignificant ( P>0.05 ) variations were detected between the broilers that did not receive honey and the broilers that did receive honey daily at the two levels. At 21 and 28 d of age, significant ( P<0.01 ) variations were detected among BW of broilers of all treatments, except for between broilers that received honey at the two levels day after day, which was insignificant. At 35 d of age, the heaviest live BW was obtained from broiler chicks that received 26 mL honey per L water a day after 2 d, followed by broilers that received 13 mL honey per L water a day after 2 d, while the lowest BW was obtained from broiler chicks that did not receive honey.

**Table 4:** Effect of honey supplementation in drinking water on weekly body weight and weight gain of broiler chickens.

		Body weight (g)						Body weight gain (g)						Viability (%)
Treatments		Period (days)												
		1	7	14	21	28	35	1–7	7–14	14–21	21–28	28–35	1–35	1–35
Control		50.5	159.0^c^	438.7^d^	800.0^f^	1330.0^f^	1700.0^g^	109.0^c^	280.4^b^	361.3^f^	530.0^e^	370.0^g^	1649.5^g^	93.33^b^
13 mL honey	Daily	50.3	160.5^c^	440.4^d^	826.3^e^	1420.0^e^	1800.0^f^	110.2^c^	279.9^b^	385.9^e^	593.9^d^	380.0^f^	1749.7^f^	97.33^ab^
per L	Day after day	50.4	165.0^bc^	448.5^bc^	872.0^c^	1480.0^c^	1900.0^d^	114.6^bc^	283.5^b^	423.5^c^	608.0^c^	420.0^d^	1849.6^d^	96.00^ab^
	Day after 2 d	50.3	185.0^a^	484.0^a^	938.9^b^	1560.0^b^	1990.0^b^	134.7^a^	299.0^a^	454.9^b^	621.1^b^	430.0^b^	1939.7^b^	97.33^ab^
26 mL honey	Daily	50.3	162.5^c^	444.0^cd^	850.0^d^	1450.0^d^	1850.0^e^	112.2^c^	281.5^b^	406.0^d^	600.0^cd^	400.0^e^	1799.7^e^	100^a^
per L	Day after day	50.4	170.0^b^	454.0^b^	880.0^c^	1490.0^c^	1960.0^c^	119.6^b^	284.0^b^	426.0^c^	610.0^c^	470.0^c^	1909.6^c^	97.33^ab^
	Day after 2 d	50.2	187.0^a^	488.0^a^	980.0^a^	1650.0^a^	2130.0^a^	136.8^a^	301.0^a^	492.0^a^	670.0^a^	480.0^a^	2079.8^a^	97.33^ab^
P value		0.865	0.011	0.005	0.004	0.001	0.001	0.004	0.008	0.004	0.008	0.005	0.001	0.04
SEM		0.20	1.85	2.5	1.75	6.10	3.45	1.95	1.45	2.50	3.10	2.89	8.79	1.30

Means of each column followed by the same letter are not significantly different at the 1 % level according to Duncan's multiple range test.

During all periods, broilers received honey at 13 or 26 mL per L water day after 2 d significantly ( P<0.01 ) surpassed BWG compared to broilers that received honey daily day after day or did not receive honey. During 1–7 and 7–14 d of age, a significant ( P<0.01 ) increase in BWG was observed in broilers that received honey day after 2 d, either at a level of 13 or 26 mL per L water compared with other treatments, whereas an insignificant ( P>0.05 ) increase was detected in broilers that received honey daily at the two levels compared with broilers in the control group. During 28–35 and 1–35 d of age, significant ( P<0.01 ) variations were detected between all groups (Table 4).

Compared to the viability rate (93.33 %) in control birds, the viability rate reached 97.33 % in birds that received honey at levels of 13 mL per L water daily or day after 2 d, and 26 mL per L water day after day or day after 2 d, while it reached 100 % in birds that received a level of 26 mL per L water daily. Significant ( P<0.05 ) variations were observed between viability rates in control birds and birds receiving honey at 26 mL per L water daily. Meanwhile, insignificant differences were found among birds receiving other honey levels and control birds, and among birds receiving honey at any level (Table 4).

The data in Table 5 display the effectiveness of honey supplementation in DW on FC, FCR, and water consumption. During 14–21, 28–35, and 1–35 d, FC was significantly ( P<0.01 ) decreased in broilers that received honey at a level of 26 mL per L water daily compared to broilers that received honey at a level of 13 mL per L water or did not receive honey. Compared to broilers that did not receive honey during experimental periods, the FCR ( P<0.01 ) improved in broilers receiving honey at 13 or 26 mL per L water. Moreover, the FCR in broilers that received honey at a level of 26 mL per L water day after 2 d significantly ( P<0.01 ) improved in comparison with broilers that received honey at a level of 26 mL per L water daily and day after day or at a level of 13 mL per L water at any time. Compared with control birds, water consumption significantly ( P<0.01 ) increased in broilers that received honey; the highest water intake was obtained in broilers that received honey daily. The water / feed ratio ranged from 1.6 to 1.87 depending on the honey rate in DW and the addition period.

**Table 5:** Effect of honey supplementation in drinking water on the feed intake, feed conversion ratio, and water intake of broiler chicks.

		Feed intake (g)						Feed conversion ratio ( $g\;g^{-1}$ )						Water intake (mL per bird)	Water / feed ratio
Treatments		Period (days)													
		1–7	7–14	14–21	21–28	28–35	1–35	1–7	7–14	14–21	21–28	28–35	1–35	1–35	
Control		150.0	395.6^a^	587.9^a^	890.9^a^	941.4^a^	2965.8^a^	1.38^a^	1.41^a^	1.63^a^	1.68^a^	2.54^a^	1.80^a^	4745.37^d^	1.60^d^
13 mL honey	Daily	146.6	375.2^cd^	570.1^b^	875.2^bc^	919.9^bc^	2880.2^de^	1.33^b^	1.34^b^	1.48^b^	1.47^b^	2.42^b^	1.65^b^	5395.72^a^	1.87^a^
per L	Day after day	147.9	384.1^bc^	581.1^a^	887.3^a^	926.3^bc^	2926.7^bc^	1.29^c^	1.35^b^	1.37^c^	1.46^b^	2.2^d^	1.58^c^	5238.88^b^	1.79^b^
	Day after 2 d	149.2	391.5^ab^	585.3^a^	889.3^a^	930.4^ab^	2945.7^ab^	1.11^e^	1.31^c^	1.29^d^	1.43^b^	2.16^e^	1.52^cd^	4978.33^c^	1.69^c^
26 mL honey	Daily	146.2	368.0^d^	545.0^d^	868.4^c^	900.9^d^	2835.3^f^	1.30^c^	1.31^c^	1.34^e^	1.45^b^	2.25^c^	1.58^c^	5273.79^b^	1.86^a^
per L	Day after day	146.9	372.0^d^	551.3^cd^	880.1^ab^	912.7^cd^	2863.0^e^	1.23^d^	1.31^c^	1.29^e^	1.44^b^	1.94^f^	1.50^d^	5153.57^b^	1.80^b^
	Day after 2 d	148.5	385.5^b^	559.5^c^	885.5^ab^	923.8^bc^	2902.8^cd^	1.09^f^	1.28^d^	1.14^f^	1.32^c^	1.92^f^	1.40^e^	4963.86^c^	1.71^c^
P value		0.521	0.002	0.004	0.001	0.003	0.004	0.001	0.001	0.001	0.001	0.001	0.001	0.007	0.004
SEM		1.50	2.75	2.43	3.13	4.98	7.65	0.01	0.01	0.01	0.01	0.02	0.02	30.64	0.01

Means of each column followed by the same letter are not significantly different at the 1 % level according to Duncan's multiple range test.

### Carcass traits

As shown in Table 6, broilers receiving honey at a level of 26 mL per L water day after 2 d reflected a significant ( P<0.01 ) increase in the relative weight of carcass and liver weight comparison with broilers receiving honey (13 $\mathrm{mL}\;L^{-1}$ ) daily or not receiving honey. Also, broilers that received honey at a level of 26 mL per L water day after 2 d showed a significant ( P<0.01 ) increase in the relative weight of the heart and thymus in comparison with broilers that received honey (13 $\mathrm{mL}\;L^{-1}$ ) daily and day after day, or (26 $\mathrm{mL}\;L^{-1}$ ) daily or did not receive honey. In addition, supplementation in DW (26 mL per L water day after 2 d) significantly ( P<0.01 ) increased the relative weight of the bursa and spleen compared with broilers receiving honey daily, day after day, or did not receive honey. Meanwhile, insignificant differences were found among broilers that received honey and did not receive it for the relative weight of gizzard honey. Compared to broilers deprived of honey, the broilers receiving honey at a level of 26 mL per L water day after 2 d showed an increase in the carcass, gizzard, liver, heart, and thymus of 10.81, 3.28, 18.00, 43.33, and 38.46, respectively. Spleen weight increased from 0.06 % to 0.11 %, representing an absolute change of 0.05 percentage points.

**Table 6:** Effect of honey supplementation in drinking water on the relative weight of the carcass and organs of broiler chickens at 35 d of age.

Treatments		Relative to pre-slaughter weight (%)						
		Carcass	Gizzard	Liver	Heart	Thymus	Bursa	Spleen
Control		70.66^c^	1.22	2.00^c^	0.30^d^	0.13^d^	0.02^e^	0.06^e^
13 mL honey	Daily	72.09^bc^	1.23	2.23^b^	0.33^cd^	0.14^cd^	0.03^d^	0.07^d^
per L	Day after day	74.81^ab^	1.24	2.26^ab^	0.36^bc^	0.15^bc^	0.04^c^	0.07^d^
	Day after 2 d	76.60^a^	1.25	2.29^ab^	0.39^ab^	0.16^ab^	0.05^b^	0.08^c^
26 mL honey	Daily	75.19^ab^	1.24	2.30^ab^	0.37^bc^	0.15^bc^	0.04^c^	0.09^b^
per L	Day after day	77.30^a^	1.25	2.34^a^	0.40^ab^	0.17^a^	0.05^b^	0.09^b^
	Day after 2 d	78.30^a^	1.26	2.36^a^	0.43^a^	0.18^a^	0.07^a^	0.11^a^
P value		0.001	0.546	0.001	0.004	0.008	0.004	0.008
SEM		1.26	0.01	0.02	0.01	0.01	0.01	0.01

Means of each column followed by the same letter are not significantly different at the 1 % level according to Duncan's multiple range test.

### Hematological parameters

The impact of honey supplementation in DW on broilers' hematological traits is displayed by the data in Table 7. In comparison with broilers that did not receive honey, an insignificant increase of Hb, eosinophils, and heterophils (H); and a significant ( P<0.05 ) increase in WBCs, lymphocytes (L), and monocytes ( P<0.01 ) were detected in the blood of broiler chickens that received honey. Furthermore, significant ( P<0.05 ) increases of RBCs and PCV were observed in the blood of broilers receiving honey at 26 mL per L water compared with broilers receiving honey at a level of 13 mL per L water or did not receive honey. In contrast, a significant ( P<0.01 ) decrease of basophils in the blood of broiler chickens received honey at a level of 13 or 26 mL per L water daily compared with other treatments. Also, a significant ( P<0.05 ) reduction of H / L ratio (5.66 %) in the blood of broiler chickens received honey at a level of 26 mL per L water day after 2 d compared to control birds.

**Table 7:** Effect of honey supplementation in drinking water on hematological parameters of broiler chickens at 35 d of age.

Treatments		RBC	Hb	PCV	WBC	Monocytes	Basophils	Eosinophils	Heterophils	Lymphocytes	H / L ratio
		( $\times 10^{6}$ per 100 mL )	(g per 100 mL )	(%)	( $\times 10^{3}$ per 100 mL )	(%)	(%)	(%)	(%)	(%)	
Control		12.70^b^	11.00	30.04^b^	23.00^b^	2.60^b^	0.85^a^	2.75	21.00	39.5^b^	0.53^a^
13 mL honey	Daily	14.0^ab^	11.70	33.21^ab^	25.40^a^	3.01^a^	0.68^b^	2.82	22.50	43.00^a^	0.52^ab^
per L	Day after day	14.10^ab^	11.60	32.50^ab^	25.35^a^	3.12^a^	0.82^a^	2.78	22.30	42.50^a^	0.52^ab^
	Day after 2 d	13.30^ab^	11.40	32.01^ab^	25.32^a^	3.12^a^	0.83^a^	2.77	22.20	42.30^a^	0.52^ab^
26 mL honey	Daily	14.90^a^	12.20	34.90^a^	25.50^a^	3.09^a^	0.67^b^	2.83	21.50	43.04^a^	0.50^b^
per L	Day after day	14.0^a^	11.90	34.50^a^	25.49^a^	3.13^a^	0.83^a^	2.83	21.10	42.50^a^	0.50^b^
	Day after 2 d	14.60^a^	11.90	34.03^a^	25.47^a^	3.13^a^	0.84^a^	2.80	21.06	42.50^a^	0.50^b^
P value		0.021	0.658	0.041	0.022	0.001	0.001	0.896	0.865	0.032	0.023
SEM		0.40	0.45	0.64	0.77	0.06	0.01	0.04	0.64	0.87	0.01

Means of each column followed by the same letter are not significantly different at the 5 % level according to Duncan's multiple range test. RBC = red blood cell, Hb = hemoglobin, PCV = packed cell volume, WBC = white blood cell, H / L = heterophils / lymphocytes ratio.

Compared to broilers that did not receive honey, the broilers that received honey at a level of 26 mL per L water day after 2 d showed an increase in RBCs, HB, PCV, WBCs, monocytes, and lymphocytes of 14.96 %, 8.18 %, 13.28 %, 10.74 %, 20.38 %, and 7.59 %, respectively.

### Blood constituents 

The data presented in Table 8 summarize how honey supplementation in DW affected the blood constituents and lipid profile. The largest values of total protein, albumin, globulin, and HI titer were obtained from chicks that received honey at 26 mL per L water daily, followed by chicks that received 26 mL honey per L water day after day. In contrast, the lowest values were obtained from chicks that did not receive honey. Significant ( P<0.01 ) differences between broilers receiving and not receiving honey were detected for globulin, albumin, and total protein, with insignificant differences among broilers that received honey at any time and at any level. Also, significant ( P<0.01 ) differences between broilers that received and did not receive honey were detected for HI titer, with significant ( P<0.01 ) differences between broilers that received 13 and 26 mL honey per L water.

**Table 8:** Effect of honey supplementation in drinking water on blood constituents and hemagglutination inhibition (HI) of broiler chickens at 35 d of age.

Treatments		Total protein	Albumin	Globulin	Uric acid	Creatinine	Triglycerides	Cholesterol	AST	ALT	HI
		( $g\;{\mathrm{dL}}^{-1}$ )	( $g\;{\mathrm{dL}}^{-1}$ )	( $g\;{\mathrm{dL}}^{-1}$ )	( $\mathrm{mg}\;{\mathrm{dL}}^{-1}$ )	( $\mathrm{mg}\;{\mathrm{dL}}^{-1}$ )	( $\mathrm{mg}\;{\mathrm{dL}}^{-1}$ )	( $\mathrm{mg}\;{\mathrm{dL}}^{-1}$ )	( $U\;L^{-1}$ )	( $U\;L^{-1}$ )	(log^2^)
Control		5.60^b^	3.00^b^	2.60^b^	5.00^a^	1.39^a^	186.00^a^	218.20^a^	60.14	67.10^a^	3.00^e^
13 mL honey	Daily	6.20^a^	3.20^a^	3.00^a^	3.51^b^	1.30^b^	182.30^b^	212.50^bc^	59.60	65.12^b^	3.70^c^
per L	Day after day	6.18^a^	3.17^a^	3.01^a^	3.42^b^	1.23^c^	183.30^ab^	213.30^b^	59.54	63.72^bcd^	3.60^c^
	Day after 2 d	6.17^a^	3.16^a^	3.01^a^	3.31^bc^	1.22^cd^	184.40^ab^	213.59^b^	57.90	62.70^cd^	3.40^d^
26 mL honey	Daily	6.30^a^	3.23^a^	3.07^a^	3.20^cd^	1.21^cd^	181.50^b^	208.30^cd^	59.19	64.04^cb^	4.30^a^
per L	Day after day	6.28^a^	3.22^a^	3.06^a^	3.12^cd^	1.20^d^	182.50^b^	209.50^cd^	58.56	63.0^cd^	4.20^a^
	Day after 2 d	6.26^a^	3.21^a^	3.05^a^	3.05^d^	1.11^e^	183.10^b^	210.30^d^	58.28	62.79^d^	3.90^b^
P value		0.002	0.001 >	0.001 >	0.001 >	0.001 >	0.026	0.001 >	0.865	0.001 >	0.001 >
SEM		0.02	0.01	0.02	0.03	0.01	0.92	0.98	0.68	0.60	0.06

Means of each column followed by the same letter are not significantly different at the 5 % level according to Duncan's multiple range test. AST = aspartate aminotransferase, ALT = alanine aminotransferase, and HI = hemagglutination inhibition.

In contrast, honey supplementation in DW (13 or 26 mL per L water) significantly ( P<0.01 ) declined cholesterol, triglycerides, creatinine, uric acid, and ALT. Insignificant ( P>0.05 ) reduction of AST occurred in broilers that received honey at the two levels at any time compared to broilers that did not receive honey. Compared to broilers deprived of honey, the broilers that received honey at a level of 26 mL per L water day after 2 d showed an increase in total protein, albumin, globulin, and HI of 11.79 %, 7.00 %, 17.31 %, and 30.00 %, respectively. At the same time, a reduction occurred in uric acid, creatinine, triglycerides, cholesterol, and ALT of 39.00 %, 20.14 %, 1.56 %, 3.62 %, and 6.42 %, respectively.

## 4 Discussion

The contents of moisture, sucrose, glucose, and fructose in Egyptian clover honey were within the acceptable range of the allowed range of Codex Alimentarius (Codex Alimentarius Commission, 2001). The amount of crude protein and ash in Egyptian clover honey was within the range of honey from Brazil (Azeredo et al., 2003), honey from the United Arab Emirates (Habib et al., 2014), honey from Egypt (Taha and El-Sanat, 2007), and honey from Tunisia (Boussaid et al., 2018). These compositional similarities indicate that the nutritional and biochemical profile of our honey is typical of high-quality multifloral honey, which is known to contain enzymes, organic acids, amino acids, minerals, and a rich matrix of bioactive compounds (Japhet and Oyingebarakumo, 2021). The amount of protein, ash, and antioxidants in honey may contribute to the content and the origin of the pollen (Taha, 2015; Al-Kahtani and Taha, 2020; Al-Kahtani et al., 2020; Taha and Al-Kahtani, 2020; Taha et al., 2024) in the honey. The values of pH and acidity of our honey were within the permitted range – pH 3.40–6.10 and ≤50 $\mathrm{mmol}\;{\mathrm{kg}}^{-1}$ , respectively – of the Codex Alimentarius (Codex Alimentarius Commission, 2001). The high acidity resulted from honey sugar fermentation into organic acid (Bogdanov et al., 2008). Total phenols in the tested honey were nearly like that of Egyptian honey (Taha and El-Sanat, 2007). Phenolic compounds scavenge the free radicals by donating hydrogen atoms or electrons due to phenolic hydroxyl group occurrence (Casquete et al., 2015). In addition, phenolic acid, flavonoids, and simple phenols possess pharmacological effects (Shara and Stohs, 2015). Moreover, phenolic compounds had antimicrobial and antioxidant activities (Farag et al., 2024). These biochemical properties provide a mechanistic basis for many of the physiological responses observed in honey-supplemented birds.

The live BW, BWG, and FCR were significantly improved in broiler chicks receiving honey in DW, supporting the findings of Japhet and Oyingebarakumo (2021) and Cai et al. (2022). Mechanistically, honey contains readily absorbable monosaccharides (glucose and fructose), digestive enzymes (invertase, diastase), and prebiotic oligosaccharides, which together enhance intestinal digestion and nutrient assimilation. Fructose is efficiently utilized by the liver for energy metabolism, while glucose supports rapid cellular ATP generation, which may explain the improved growth performance in younger birds that have higher metabolic demands (Ahmed et al., 2012). Additionally, honey flavoring has been shown to improve feed palatability and stimulate appetite (Obun et al., 2010). The marked improvements in growth performance observed in the present study may be attributed to the honey-mediated enhancement of gut microflora, increased activity of fibrolytic enzymes, and improved intestinal morphology, as previously suggested by Cai et al. (2022) and Hegazi et al. (2013).

Furthermore, the presence of diastase and invertase enzymes in honey facilitates the hydrolysis of complex carbohydrates into simple sugars, providing an immediate energy source that improves feed efficiency (Mandal et al., 2004). Honey also contains essential minerals (K, Ca, P, Mg, Fe, Zn, Se, Cr, Mn), vitamins (pantothenic acid, niacin, riboflavin, folic acid, vitamin B6, ascorbic acid), and phenolic compounds (Taha et al., 2021; Ball, 2007), all of which play a role in metabolism, antioxidant protection, and immune regulation. Islam et al. (2017) reported that honey contains about 0.1 % polyphenols (equivalent to 296 mg gallic acid), and these compounds have been shown to increase serum antioxidant enzyme activity in broilers (Farag et al., 2024). Therefore, the superior performance of honey-supplemented birds in our study is likely mediated through a combination of improved energy utilization, enhanced digestive efficiency, and a reduction in oxidative stress.

Our findings showed that FC was significantly depressed in broiler chicks that received honey in DW. These findings partially support the findings of Jimoh et al. (2017), who reported that honey supplementation significantly reduced feed intake. The reduction in FCR in broiler chicks that received honey may be attributed to high levels of fructose (40.44 %) and glucose (32.11 %) in honey. The decrease in FC alongside improved FCR suggests more efficient nutrient use, possibly due to enhanced glucose absorption, improved gut motility, and the prebiotic action of honey's oligosaccharides. Simple sugars in honey are absorbed directly into the bloodstream and result in high levels of sugar in the blood, which may signal systematizing FCR by the birds (Jeffrey and Echazarreta, 1996). The positive effects on FCR most likely stem from a synergistic interaction between the physiological advantages of increased water intake and the bioactive ingredients of honey, including its sugars, enzymes, and prebiotic oligosaccharides (Abd-El-Aziz et al., 2023). It is possible that the honey solution's palatability encouraged higher water consumption, which in turn encouraged more effective feed utilization (Bogdanov et al., 2008). In contrast, honey supplementation had an insignificant impact on FI, WG, and FCR (Abioja et al., 2019).

Water is an essential component in poultry nutrition and plays a part in feed digestion and absorption, nutrient transportation, waste disposal (Williams et al., 2013), and thermal homeostasis. It also constitutes 70 %–80 % of live body mass in birds (Salas et al., 2012). Daily water intake is a good test of the overall health and condition of the birds (Manning et al., 2007). Our water / feed ratios were within the previous ratios (1.5–2.0) obtained by Georgia (2001) and Obaia (2015). The increased water intake in honey groups may have contributed to improved nutrient solubilization and gut transit, indirectly supporting better growth performance (Abd El-Aziz et al., 2023).

The viability percentage increased noticeably in broilers receiving honey in their drinking water. This improved survival is likely linked to honey's antimicrobial, antioxidant, and immunostimulatory properties. Its phenolic compounds and polyphenols can suppress harmful bacteria such as *Staphylococcus aureus* and *Escherichia coli* while supporting beneficial gut microflora (Farag et al., 2024), thereby reducing disease pressure and strengthening overall resilience. These findings align with Obun et al. (2010), who reported no mortality in birds supplemented with 1.0 % honey.

Honey's antimicrobial activity is primarily attributed to its low pH, high osmotic pressure, hydrogen peroxide content, and diverse phenolic compounds, all of which work together to suppress pathogenic bacteria (Molan and Betts, 2004). These bioactive components disrupt microbial cell walls, inhibit bacterial growth, and support the proliferation of beneficial gut microflora. Such effects help to reduce enteric infections and overall disease pressure, contributing to improved survival and health status in broiler chickens (Bogdanov et al., 2008).

In addition, honey provides strong antioxidants and immunostimulatory benefits through its rich content of flavonoids, phenolic acids, enzymes, and vitamins. These compounds neutralize free radicals, minimize oxidative stress, and help to protect tissues during rapid growth or environmental challenges. Honey also enhances immune responsiveness by stimulating lymphoid organs, boosting antibody production, and modulating cytokine activity. Together, these antioxidant and immune-enhancing effects contribute to better physiological resilience, improved performance, and reduced mortality in honey-supplemented broilers (Japhet and Oyingebarakumo, 2021; Abd El-Aziz, 2023).

Honey supplementation in DW at a rate of 26 mL per L water per day after 2 d led to a significant increase in the relative weight of the carcass, liver, bursa, spleen, heart, and thymus compared with those that did not receive honey. Meanwhile, a slight increase occurred in the gizzard weight of broilers that received honey. Our results agree with those of Abioja et al. (2019) for the thymus and gizzard, while the heart, liver, bursa, and spleen are insignificantly influenced by honey supplementation. Moreover, Obun et al. (2010) reported that the liver, heart, and gizzard weights are numerically increased by increasing the honey flavor in diets. In addition, the weight of lymphoid organs was improved in honey-supplemented birds (Cai et al., 2022; Memon et al., 2019). The improvement of the previous organs may be due to the biological compound in honey. The biologically active component in honey is partially related to its pollen content (Al Naggar et al., 2024). Honey may increase lymphoid organ weights by supplying flavonoids, phenolic acids, and pollen-derived phytochemicals that actively support immune system development. These bioactive compounds can modulate cytokine expression, enhance the maturation and activity of immune cells, and stimulate lymphocyte proliferation, all of which promote greater growth and functional capacity of organs such as the thymus, spleen, and bursa of Fabricius. As these organs expand and become more active, birds exhibit stronger immune responsiveness and improved overall resilience (Kamboh et al., 2018).

The hematological analysis of broilers varied significantly and depended on the honey supplemented to the broiler chicks. In the current work, honey supplementation in DW significantly increased PCV, RBCs, WBCs, monocytes, and lymphocytes compared to the broilers deprived of honey. In contrast, daily honey supplementation in DW significantly reduced the concentration of basophils. In addition, a significant reduction in the H / L ratio occurred in broilers receiving honey at 26 mL per L water. The current findings confirm the results of Abioja et al. (2019), as they found that broilers fed a diet containing corticosterone at 30 $\mathrm{mg}\;{\mathrm{kg}}^{-1}$ concentration and receiving honey at levels of 5, 10, or 15 mL per L DW for 7 d had a significant increase in PCV, RBCs, and HB compared to those in the control group. On the other hand, basophil, monocyte, eosinophil, lymphocyte, heterophil, and H / L ratio were insignificantly influenced when honey was added to DW for heat-stressed birds (Akibo, 2006). Also, the insignificant impact of honey on PCV, HB, total WBC and RBC count, and differential leukocyte count was recorded by Oke et al. (2016). Furthermore, it was observed that there were insignificant differences in WBCs, basophil, monocyte, lymphocyte, heterophil, and the H / L ratio of broiler chickens receiving honey at levels of 5, 10, or 15 mL per L DW for 7 d and control (Abioja et al., 2019). These hematological improvements reflect enhanced erythropoiesis, immune cell proliferation, and reduced physiological stress. The elevation in RBCs and PCV may be linked to honey's iron, copper, and folate content, which supports hemoglobin synthesis (Oke et al., 2016).

Honey supplementation (26 mL honey per L water) significantly raised the total protein, albumin, and globulin. Similar findings were reported by Obun et al. (2008) for total protein, albumin, and creatinine. In addition, Oke et al. (2016) reported similar results for total protein, albumin, globulin, and ALT; while Hamed et al. (2019) observed comparable effects on total protein, globulin, triglycerides, and ALT. Meanwhile, Abioja et al. (2019) recorded that broiler chickens' globulin, albumin, total protein, uric acid, creatinine, and ALT have not been significantly influenced by supplemented honey in DW. In contrast, triglyceride concentration increased in broiler chicks that received DW with honey at 60 $\mathrm{mL}\;L^{-1}$ , which was significantly higher than in broilers that received honey at a level of 20 and 40 $\mathrm{mL}\;L^{-1}$ (Oke et al., 2016). The increase in serum proteins and globulins may indicate enhanced hepatic protein synthesis and improved humoral immunity, consistent with the increased lymphoid organ weights. The reductions in serum lipids and ALT suggest that honey positively influences lipid metabolism and liver function through its antioxidant and hepatoprotective components (Obun et al., 2008; Hamed et al., 2019).

Birds supplemented with honey also exhibited higher antibody titers against Newcastle disease virus, reflecting an enhanced immune status. This improvement is likely due to honey's rich content of polyphenols, minerals (such as zinc and selenium), and vitamins (including vitamin C and vitamin B complex), which collectively support antibody synthesis and stimulate lymphocyte activity (Bogdanov et al., 2008). Similarly, Japanese quail receiving honey (22 $g\;L^{-1}$ ) showed elevated Newcastle disease antibody levels compared with both virginiamycin-treated and control groups (Babaei et al., 2016).

## 5 Conclusions

In conclusion, honey supplementation via drinking water at 26 $\mathrm{mL}\;L^{-1}\;d^{-1}$ after 2 d significantly enhanced growth performance, carcass characteristics, hematological traits, and hemagglutination inhibition. In addition, honey supplementation reduces serum triglycerides, cholesterol, alanine aminotransferase, creatinine, and uric acid.

## Data Availability

The data presented in this study are available free of charge for any user upon reasonable request from the corresponding author.
